# Adrenal ganglioneuroma: two case reports and literature review

**DOI:** 10.3389/fonc.2025.1619030

**Published:** 2025-07-29

**Authors:** Zhenzhou Gao, Haokai Qin, Bao Li, Hongjun Zhao, Mingqing Zhang

**Affiliations:** ^1^ School of Clinical Medicine, Shandong Second Medical University, Weifang, Shandong, China; ^2^ Department of Urinary Surgery, Weifang People’s Hospital, Weifang, Shandong, China

**Keywords:** adrenal glands, ganglioneuroma, necrosis, hypertension, angiomatous component, immunohistochemistry

## Abstract

Adrenal Ganglioneuroma (AGN) is a rare benign tumor that is mostly nonfunctioning and usually presents without significant clinical symptoms. AGN can undergo calcification, but rarely presents with hemorrhage, necrosis, or cystic changes. This article reports two cases of adrenal ganglioneuroma. One patient had normal clinical and biochemical parameters but presented with hypertension; the other patient had elevated cortisol levels and the tumor exhibited liquefactive necrosis, which is extremely rare in AGN. To the best of our knowledge, this is the first report of adrenal ganglioneuroma with simultaneous localized hemangiomatous proliferation and liquefactive necrosis within the tumor. AGN cannot be definitively diagnosed by imaging alone; a pathological examination is required for a final diagnosis. Both patients in this report underwent laparoscopic tumor resection, and their postoperative recovery was satisfactory.

## Introduction

Ganglioneuroma (GN) is a rare benign tumor that grows slowly and originates from the primitive sympathetic nerves (1). It is mainly composed of mature ganglion cells, Schwann cells, and nerve fibers (2). GN can occur in various parts of the body, most commonly in the retroperitoneum (32–52%) and posterior mediastinum (39–43%), followed by the neck (8–9%), and its occurrence in the adrenal gland and intestinal tract is relatively rare (3, 4). Some reports have noted that GN may cause erosion of the adjacent bones during growth (5). AGN is rare in children under 10 years of age, with a higher incidence in adults than in children, and its incidence increases with age. There are no significant sex differences in morbidity (1, 3). The pathogenesis remains unclear, but abnormal expression of ERBB3 and GATA3 may be associated with its development (3). This article reports a case of AGN with special clinical manifestations and one case of AGN with rare endocrine and pathological features, with the aim of enhancing the understanding of AGN to improve diagnostic and therapeutic outcomes.

## Case 1

**Figure 1:**
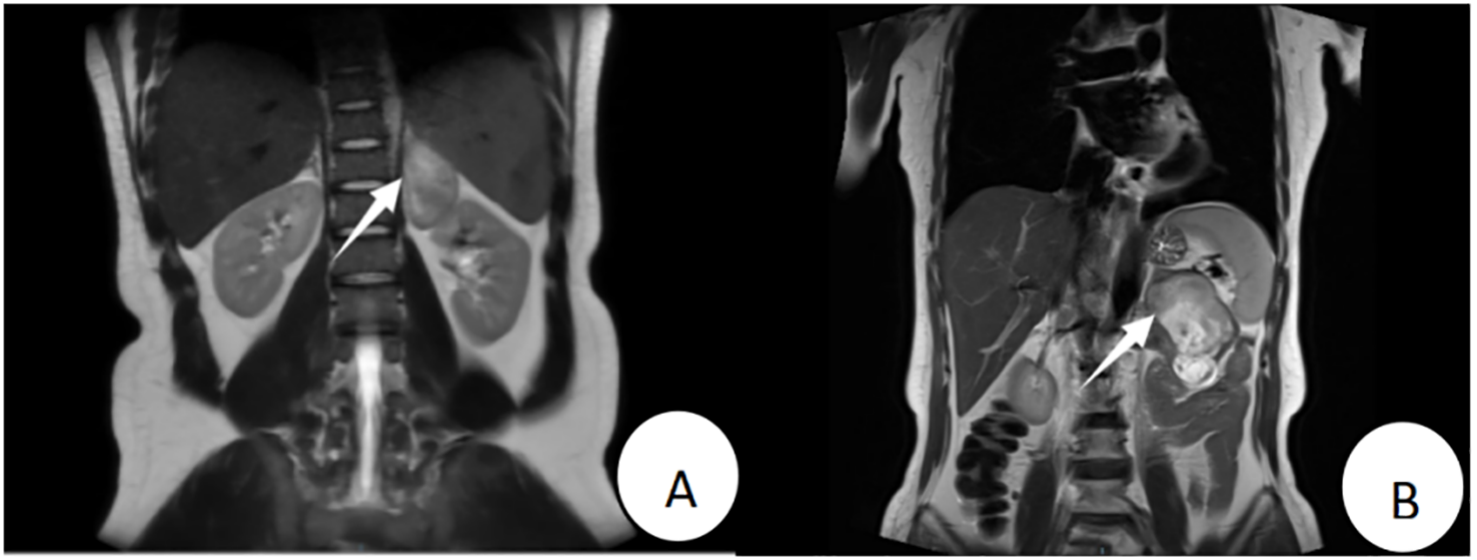
Patient 1’s preoperative MRI (T2WI) revealed a mass of approximately 7.5 cm × 7.0 cm × 3.5 cm in the left adrenal region **(A)**; Patient 2’s preoperative MRI (T2WI) showed a mass of approximately 9.0 cm × 5.5 cm × 4.8 cm anterior to the left adrenal region **(B)**.

**Figure 2:**
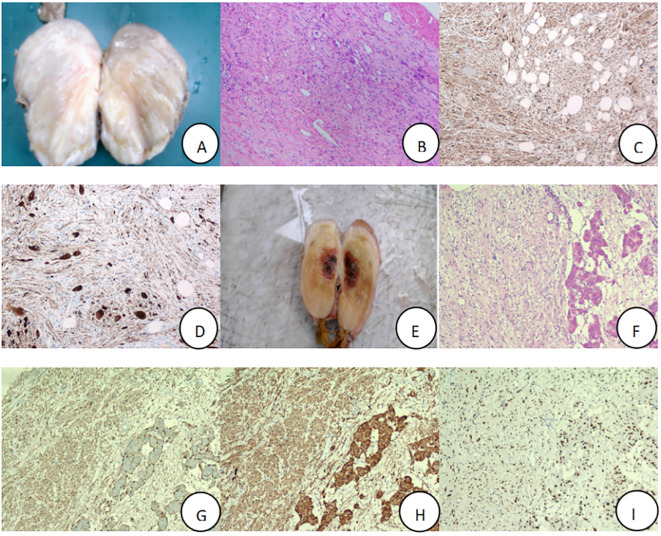
Postoperative specimens of adrenal ganglioneuroma, with routine pathology and immunohistochemistry (×100), Patient 1:tumor tissue **(A)**, Hematoxylin and eosin-stained section result **(B)**, S-100 (+) **(C)**, Syn (+) **(D)**; Patient 2: tumor tissue **(E)**, Hematoxylin and eosin-stained section result **(F)**, S-100 (+) **(G)**, Syn (+) **(H)**, SOX10 (+) **(I)**.

The patient, a 22-year-old male, was admitted due to “elevated blood pressure for more than 5 months and detection of a left adrenal mass during imaging examination for 7 days,” accompanied by dizziness, headache, palpitations, and chest tightness. He had a 5-month history of hypertension with a maximum blood pressure of 165/110 mmHg. He was taking oral metoprolol tartrate (1 tablet/day) and amlodipine besylate (1 tablet/day), and his blood pressure was well-controlled. Physical examination revealed soft abdomen, no tenderness or rebound tenderness, no palpable mass, and no tenderness or percussion pain in the renal or ureteral regions. After admission, his blood pressure was measured at 126/78 mmHg. Bilateral adrenal computed tomography (CT) revealed a mass in the left adrenal region measuring approximately 7.5 cm × 7.0 cm × 3.5 cm, with relatively homogeneous texture. Bilateral adrenal magnetic resonance imaging (MRI) revealed a mass in the left adrenal region, isointense on T1WI and mixed hyperintensity on T2WI, with no obvious diffusion restriction. The lesion measured approximately 7.5 cm × 7.0 cm × 3.5 cm (**Figure 1A**). Laboratory tests revealed no abnormalities in the supine and upright hypertension panels (including renin, angiotensin, aldosterone, and cortisol), 24-Hour Urine Catecholamines Test(including dopamine, norepinephrine, and epinephrine), complete blood count, liver and renal function, or electrolytes. After ruling out contraindications for surgery, the patient underwent laparoscopic left adrenal tumor resection on the 7th day of hospitalization. Intraoperatively, the tumor had a well-defined border with no obvious adhesion to the surrounding tissues and was dissected along the outer capsule. Postoperative pathology revealed a nodular tumor measuring 7.5 cm × 7.0 cm × 3.5 cm. The cut surface was gray-white and firm, with attached adrenal tissue (**Figure 2A**). Microscopically, spindle cells were arranged in bundles and mature ganglion cells were visible (**Figure 2B**). Immunohistochemical staining revealed S-100 (+) (**Figure 2C**), Syn (+) (**Figure 2D**), Vimentin (+), Pan-CK (−), CgA (−), TH(−), PHOX2B(−), GFAP (−), NeuN (focally +), and Ki-67 index (hotspot) <1%. The pathological diagnosis was a ganglioneuroma. The patient recovered well postoperatively and was discharged on postoperative day five. At the 3-month outpatient follow-up, the patient reported no specific discomfort, and follow-up CT of both adrenal glands showed no evidence of recurrence. His blood pressure remained within the normal range without antihypertensive medications, indicating good recovery.

## Case 2

The patient, a 57-year-old female, was admitted due to”a left adrenal mass detected during imaging examination for over half a month.” She had no hypertension, palpitations, chest tightness, excessive sweating, dizziness, headache, nausea, or vomiting. She had a history of asthma for over one month. Physical examination revealed a soft abdomen without tenderness or rebound tenderness, no palpable mass, and no tenderness or percussion pain in the renal or ureteral region. After admission, a urinary system ultrasound revealed a solid lesion in the left adrenal region. Bilateral adrenal CT revealed a mass in the left adrenal region with heterogeneous density, patchy low-density, punctate high-density areas, and heterogeneous enhancement on contrast-enhanced scanning. Bilateral adrenal MRI revealed a well-defined space-occupying lesion in the anterior portion of the left adrenal region, isointense to slightly hyperintense on T1WI, mixed hyperintense on T2WI, measuring approximately 9.0 cm × 5.5 cm × 4.8 cm, with areas of liquefactive necrosis within (**Figure 1B**). Laboratory tests revealed 8am cortisol: 636.9 nmol/L (reference range 133-537 nmol/L), complete blood count, liver and renal function, electrolytes, serum renin, angiotensin, aldosterone, adrenocorticotropic hormone, and catecholamines were all within normal ranges. Negative result of the 1 mg dexamethasone suppression test. After ruling out surgical contraindications, the patient underwent laparoscopic left adrenal tumor resection on the 6th day of hospitalization. Intraoperatively, the tumor was closely associated with the left adrenal gland and dissected along the outer capsule. Postoperative pathology revealed a nodular mass measuring 9.0 cm × 5.5 cm × 4.8 cm. The cut surface was gray-yellow and firm, with attached adrenal tissue (**Figure 2E**). Spindle cells and mature ganglion cells were observed microscopically(**Figure 2F**). Immunohistochemical staining revealed S-100 (+) (**Figure 2G**), Syn (+) (**Figure 2H**), SOX10 (+)(**Figure 2I**), NSE (focal +), CK (−), CgA (ganglion cells +), CD56 (+), CD34 (vascular +), P16 (+), SMA (smooth muscle +), Desmin (smooth muscle +), and Ki-67 index (hotspot) 1%. The pathological diagnosis was ganglioneuroma with calcification and a focal hemangioma-like proliferation. The patient recovered well postoperatively and was discharged on postoperative day six. At the 3-month outpatient follow-up, the cortisol levels were normal, the patient reported no specific discomfort, and recovery was good.

## Discussion

AGN is mostly a nonfunctional tumor, with the majority of patients presenting without obvious clinical symptoms. They are often discovered incidentally during physical examinations. When a tumor grows large, it may cause localized compression symptoms such as abdominal or lumbar pain (6). In rare cases, AGN can secrete catecholamines, vasoactive intestinal peptides, testosterone, and other substances, leading to symptoms such as hypertension, excessive sweating, and nonspecific abdominal discomfort (3, 6). In Case 1, the tumor was discovered during bilateral adrenal CT examination due to hypertension lasting more than 5 months. In contrast, Case 2 had no obvious clinical symptoms and was incidentally found on chest CT performed due to coughing and dyspnea for over a month. Fan Hua et al. reported that among 80 pathologically confirmed AGN cases, 10 patients had blood pressure fluctuations and three had elevated 24-hour urinary free cortisol levels (7). The hypertension in Case 1 may be attributed to several factors. First, the tumor may have secreted small amounts of bioactive substances, such as neuropeptides, which are not routinely tested, indirectly affecting blood pressure regulation. Second, primary hypertension may have coexisted with the tumor. Third, the tumor may have had compression-related effects. Functional AGN may cause hypertension (8). Moreover, composite tumors formed by adrenal ganglioneuromas and pheochromocytomas can secrete catecholamines and their metabolites, leading to elevated blood pressure (9). Some studies have also shown that AGN tend to envelop surrounding blood vessels; when the mass is large, it may compress the renal artery, resulting in renal artery stenosis and subsequent hypertension (7, 10). Although laboratory tests for Case 1 showed no abnormalities, considering the large size of the mass and persistent hypertension, along with the postoperative pathological diagnosis of ganglioneuroma and normalization of blood pressure, hypertension was likely due to tumor compression or a mild neuroendocrine effect. Cortisol levels were elevated in Case 2. Given the large size of the mass, it is likely that the tumor compressed or stimulated the adrenal tissue to secrete more cortisol.

Ultrasound examination of AGN often shows hypoechoic or moderately echogenic solid lesions in the adrenal region. CT typically reveals a well-defined mass with slightly low and homogeneous density, with mild to moderate enhancement on contrast imaging. MRI commonly shows uniform low signal intensity on T1WI and heterogeneous high signal intensity on T2WI (7). However, these imaging features lack specificity and may overlap with those of other adrenal tumors. Large AGN often exhibit signs of compression of adjacent vascular structures on imaging but rarely invade organs or vascular structures, which is one of their characteristic imaging features (7). In addition, punctate calcifications on imaging are suggestive features of AGN (11). Some studies have also reported a swirling pattern on imaging due to the irregular interweaving of Schwann cells and nerve fibers within the tumor, which is considered a characteristic manifestation of AGN. Furthermore, AGN often shows minimal enhancement during the arterial phase and progressive enhancement during the portal and delayed phases, which is also of diagnostic value (2). The imaging findings of Case 1 were consistent with the typical features of AGN. In Case 2, calcification was observed on imaging, but the tumor showed heterogeneous density with liquefactive necrosis. Liquefactive necrosis may have been caused by an inadequate blood supply due to the large tumor size. Imaging for AGN lacks specificity and may overlap with those of other adrenal tumors. Therefore, AGN is rarely suspected preoperatively, and imaging findings alone are insufficient for definitive diagnosis.

The diagnosis and differential diagnosis of AGN rely mainly on pathological morphology and immunohistochemical examination. Under the microscope, AGN showed mature ganglion cells with granular cytoplasm, round nuclei, and prominent nucleoli. These cells were interwoven with the spindle-shaped cells within the myxoid stroma. Immunohistochemical staining typically reveals positive expression of S-100, NSE, and Syn (2, 8). AGN must be differentiated from adrenal neuroblastoma, adrenal ganglioneuroblastoma, pheochromocytoma, adrenal adenoma, adrenocortical carcinoma, or schwannoma. Neuroblastoma is primarily composed of undifferentiated or poorly differentiated neuroblasts, with a low proportion of Schwann cell stroma, a high mitotic index, frequent Homer-Wright pseudorosettes, and necrosis. Immunohistochemically, PHOX2B and NB84 show strong positivity, whereas S-100 is only focally expressed in the stroma (1). Ganglioneuroblastoma is classified into mixed and nodular types, with Schwann cell stroma accounting for ≥50% and the coexistence of neuroblasts and mature ganglion cells. PHOX2B and Synaptophysin are found in neuroblast-rich areas (1). In both Patients 1 and 2, no neuroblasts were observed on histological examination, and the Ki-67 index was ≤1%, allowing differentiation from adrenal neuroblastoma and adrenal ganglioneuroblastoma. Pheochromocytoma is a hormonally active tumor characterized by synthesis, storage, and release of catecholamines and other active substances. Clinically, it typically presents with hypertension and the classic triad of headaches, palpitations, and excessive sweating. Laboratory tests often reveal significantly elevated plasma and urinary metanephrines (12). Although the laboratory findings in Patient 1 did not suggest abnormal catecholamine secretion, a composite pheochromocytoma-ganglioneuroma could still cause similar clinical manifestations. However, the immunohistochemistry results showing CgA (−), TH (−), and PHOX2B (−) ruled out the possibility of a composite pheochromocytoma-ganglioneuroma. Adrenocortical carcinoma is a highly malignant and aggressive tumor of the adrenal cortex. Steroidogenic factor 1 (SF-1) is currently considered the most sensitive and specific biomarker for confirming adrenal cortical origin (13). Adrenal schwannomas are peripheral nerve tumor originating from Schwann cells. Its diagnosis mainly depends on characteristic histological features, including alternating Antoni A and B areas, nuclear palisading, and Verocay bodies (14), which can be easily differentiated from AGN.

Laparoscopic resection is the primary treatment for AGN. According to the study by Lei Kunyang et al. (15), laparoscopic surgery is the preferred approach for tumors with a diameter less than 6 cm, whereas open surgery may be more effective and safer for tumors larger than 6 cm or those suspected to have malignant potential. Other studies suggest that laparoscopic surgery is still applicable even when the tumor diameter exceeds 6 cm, and compared to open surgery, it offers advantages such as minimal invasiveness, less bleeding, reduced postoperative pain, and quicker recovery (7, 11). The postoperative outcomes of AGN are generally favorable with a low incidence of complications (15). In a study of 80 AGN cases, no tumor recurrence or metastasis was observed during postoperative follow-up ranging from 2 months to 35 years (7). In this study, both patients underwent laparoscopic tumor resection and showed a good postoperative recovery and prognosis.

In conclusion, adrenal ganglioneuroma is a rare benign tumor that originates from the sympathetic ganglia. Most patients with AGN are asymptomatic, and tumors are often discovered incidentally during routine examinations. Owing to its low incidence and lack of specific clinical features, preoperative diagnosis is challenging and relies on pathological examination for confirmation. Laparoscopic surgery is the main treatment for AGN, and its prognosis is generally favorable. The purpose of this case report is to enhance the understanding of AGN and explore more effective diagnostic and therapeutic approaches to achieve optimal treatment outcomes.

## Data Availability

The original contributions presented in the study are included in the article/Supplementary Material. Further inquiries can be directed to the corresponding author.
